# A note on contracts on quadratic variation

**DOI:** 10.1371/journal.pone.0174133

**Published:** 2017-03-23

**Authors:** Carl Lindberg

**Affiliations:** 1 The Second Swedish National Pension Fund, Gothenburg, Sweden; 2 Mathematical Sciences, Chalmers University of Technology, Gothenburg, Sweden; University of Rijeka, CROATIA

## Abstract

Given a Black stochastic volatility model for a future *F*, and a function *g*, we show that the price of $\frac{1}{2}\;\int_{0}^{T}g\;(t,F(t))F^{2}\;(t)\sigma^{2}\;(t)\;dt$ can be represented by portfolios of put and call options. This generalizes the classical representation result for the variance swap. Further, in a local volatility model, we give an example based on Dupire’s formula which shows how the theorem can be used to design variance related contracts with desirable characteristics.

## Introduction

The variance swap, and its derivatives such as the VIX index, have become the instruments of choice to trade a view on volatility. However, these products experience extreme volatility during market distress, and a short position then typically incurs dramatic losses. To circumvent this practical problem, we generalize in this paper the concept of the variance swap. This allows us to develop instruments which have more reasonable tail characteristics.

We assume a stochastic volatility model of Black type for a future *F*. Hence, under the risk neutral probability $P^{*}$, *F* has the dynamics

$$dF\;\left(t\right)=\sigma \;\left(t\right)F\;\left(t\right)dW\;\left(t\right).$$ (1)

The process *σ* is presumed to be adapted and mean square integrable, so there exists a unique positive continuous martingale solution *F*(*t*) to Eq (1) given an initial value *F*(0). Further, we assume for simplicity that the constant short rate *r* = 0.

Presented with a function *g*, we define the process

$$V\left(t\right)≔\frac{1}{2}\;\int_{0}^{t}g\;\left(u,F\left(u\right)\right)F^{2}\;\left(u\right)\sigma^{2}\;\left(u\right)\;du.$$ (2)

We show that the risk neutral price of *V*(*T*) can be represented by portfolios of put and call options. We give also the explicit hedging scheme for *V*. The result we obtain is a generalization of the classical representation formula for the variance swap. However, the main contribution of this note is its application to the design of variance related contracts. Particularly, we include an example where we apply Dupire’s formula in a Black local volatility model to choose a *g* such that *V* is independent of *F*. This is very different from the variance swap, which typically has dramatic increases in value during distress and a modest negative return during most other market conditions.

There is a large and growing literature which analyzes the variance risk premium and its effect on asset prices. A non-exclusive list includes [1–6], and [7]. These papers study mainly the variance risk premium on a single maturity. The term structure of variance swap rates is considered in [8] and [9]. A motivation for the definition of the VIX index, and its relation to variance swaps, is given in [10].

## The variation contract

We will denote by *variation contract* a derivative which pays *V*(*T*) defined in Eq (2) at *t* = *T*. Our motivation for this choice is that *V* is the integral of the quadratic variation of *F*, weighted by *g*. We present now the main result of the paper.

**Theorem 1** *For a function* *g*(*t*, *x*), *with* sup_*t* ∈ [0, *T*]_∫_*K*_ |*g*(*t*, *K*)|*dK* < ∞, *which is continuous in* (*t*, *x*) *and has a derivative* $g_{t}^{\prime }$ *satisfying the same conditions, the price of a variation contract*

$$\begin{array}{ccc} & & E^{*}\;\left[V(T)\right]\\ & = & \int_{K\leq F\left(0\right)}g\;\left(T,K\right)P\;\left(T,K\right)\;dK+\int_{K\geq F\left(0\right)}g\;\left(T,K\right)C\;\left(T,K\right)\;dK,\\ & & -\int_{0}^{T}\;\int_{K\leq F\left(0\right)}g_{t}^{\prime }\;\left(t,K\right)P\;\left(t,K\right)\;dKdt-\int_{0}^{T}\;\int_{K\geq F\left(0\right)}g_{t}^{\prime }\;\left(t,K\right)C\;\left(t,K\right)\;dKdt. \end{array}$$

*where* *P*(*x*, *y*) *and* *C*(*x*, *y*) *denote the price of the European put and call options with expiration* *x* *and strike price* *y* *at time* 0, *respectively. In addition, the hedging portfolio is given by*

$$-\int_{0}^{T}\;\int_{F\left(0\right)}^{F\left(t\right)}g\;\left(t,y\right)\;dydF\;\left(t\right).$$ (3)

**Proof.** We define

$$H\left(t\right)≔\int_{F\left(0\right)}^{F\left(t\right)}\left(F\;\left(t\right)-y\right)g\;\left(t,y\right)\;dy,$$ (4)

and see also that *H*(*t*) = *h*(*t*, *F*(*t*)), where

$$h\left(t,x\right)=\int_{F\left(0\right)}^{x}\left(x-y\right)g\;\left(t,y\right)\;dy.$$

Hence,

$$h_{t}^{\prime }\;\left(t,x\right)=\int_{F\left(0\right)}^{x}\;\left(x-y\right)g_{t}^{\prime }\;\left(t,y\right)\;dy,$$

$$h_{x}^{\prime }\;\left(t,x\right)=\int_{F\left(0\right)}^{x}\;g\;\left(t,y\right)\;dy,$$

and

$$h_{xx}^{\prime \prime }\;\left(t,x\right)=g\;\left(t,x\right).$$

Itô’s formula gives that

$$\begin{array}{ccc} H\left(T\right) & = & \int_{0}^{T}\;\int_{F\;\left(0\right)}^{F\left(t\right)}\left(F\left(t\right)-y\right)g_{t}^{\prime }\;\left(t,y\right)\;dydt+\int_{0}^{T}\;\int_{F\left(0\right)}^{F\left(t\right)}g\;\left(t,y\right)\;dydF\;\left(t\right)\\ & & +\frac{1}{2}\;\int_{0}^{T}g\;\left(t,F\;\left(t\right)\right)\sigma^{2}\;\left(t\right)F^{2}\;\left(t\right)\;dt. \end{array}$$

Combining this expression with *H*(*T*) from Eq (4), we see that

$$\begin{array}{ccc} & & \frac{1}{2}\;\int_{0}^{T}g\;\left(t,F\;\left(t\right)\right)\sigma^{2}\;\left(t\right)F^{2}\;\left(t\right)\;dt\\ & = & \int_{F\;\left(0\right)}^{F\left(T\right)}\left(F\left(T\right)-y\right)g\;\left(T,y\right)\;dy-\int_{0}^{T}\int_{F\left(0\right)}^{F\;\left(t\right)}\left(F\;\left(t\right)-y\right)g_{t}^{\prime }\;\left(t,y\right)\;dydt\\ & & -\int_{0}^{T}\;\int_{F\left(0\right)}^{F\left(t\right)}g\;\left(t,y\right)\;dydF\;\left(t\right). \end{array}$$ (5)

Further,

$$\begin{array}{c} \int_{F\left(0\right)}^{F\left(T\right)}\left(F\;\left(T\right)-y\right)g\;\left(T,y\right)\;dy\\ =\int_{K\leq F\left(0\right)}g\;\left(T,K\right){\left(K-F\left(T\right),0\right)}^{+}dK+\int_{K\geq F\left(0\right)}g\;\left(T,K\right){\left(F\;\left(T\right)-K,0\right)}^{+}dK, \end{array}$$

and

$$\begin{array}{c} \int_{F\left(0\right)}^{F\left(t\right)}\left(F\;\left(t\right)-y\right)g_{t}^{\prime }\;\left(t,y\right)\;dy\\ =\int_{K\leq F\left(0\right)}g_{t}^{\prime }\;\left(t,K\right){\left(K-F\;\left(t\right),0\right)}^{+}dK+\int_{K\geq F\left(0\right)}g_{t}^{\prime }\;\left(t,K\right){\left(F\;\left(t\right)-K,0\right)}^{+}dK. \end{array}$$

We note that the stochastic integral is a martingale under $P^{*}$. Hence, taking expectation of both sides of Eq (5) and applying Fubini’s theorem yields the result, since

$$C\;\left(t,K\right)=E^{*}\;\left[{\left(F\;\left(t\right)-K,0\right)}^{+}\right],$$

and

$$P\;\left(t,K\right)=E^{*}\;\left[{\left(K-F\;\left(t\right),0\right)}^{+}\right].$$

This theorem allows us to design variation constracts to have desirable features. For example, if we choose some optimality condition, we can optimize *V* over all feasible functions *g*. Further, and more importantly, the theorem shows explicitly how to hedge the variation contract with options portfolios which are static once initiated, and a self-financing futures portfolio. The fact that the hedging option portfolios are static is very important from an applied perspective. The weak convergence results required to analyze the hedging error associated with the implementation of *V* are given in [11].

### Example

We assume a Black local volatility model, with a sufficiently regular *σ* (*t*, *F*). If we choose *g* such that *g* (*t*, *F* (*t*)) = 2/(*σ* (*t*, *F* (*t*)) *F* (*t*))^2^ we get that *V* (*t*) = *t*. The return of this variation swap will be independent of *F*, in contrast to the return of a variance swap. The local volatility function *σ* can be derived from market prices using Dupire’s well known formula

$$\sigma^{2}\;\left(t,x\right)=\frac{2C_{T}\;\left(t,x\right)}{x^{2}C_{KK}\;\left(t,x\right)}.$$

Hence, we have that

$$g\;\left(t,F\;\left(t\right)\right)=\frac{C_{KK}\;\left(t,F\;\left(t\right)\right)}{C_{T}\;\left(t,F\;\left(t\right)\right)},$$

and the hedging portfolio is given by Eq (3).
